# The Role of Temporal and Spatial Attention in Size Adaptation

**DOI:** 10.3389/fnins.2020.00539

**Published:** 2020-06-03

**Authors:** Alessia Tonelli, Arezoo Pooresmaeili, Roberto Arrighi

**Affiliations:** ^1^Department of Translational Research of New Technologies in Medicine and Surgery, University of Pisa, Pisa, Italy; ^2^Uvip, Unit for Visually Impaired People, Istituto Italiano di Tecnologia, Genoa, Italy; ^3^Perception and Cognition Group, European Neuroscience Institute, Göttingen, Germany; ^4^Department of Neuroscience, Psychology, Pharmacology and Child Health (NEUROFARBA), University of Florence, Florence, Italy

**Keywords:** size perception, visual adaptation, spatial attention, multiple object tracking, rapid serial visual presentation

## Abstract

One of the most important tasks for the visual system is to construct an internal representation of the spatial properties of objects, including their size. Size perception includes a combination of bottom-up (retinal inputs) and top-down (e.g., expectations) information, which makes the estimates of object size malleable and susceptible to numerous contextual cues. For example, it has been shown that size perception is prone to adaptation: brief previous presentations of larger or smaller adapting stimuli at the same region of space changes the perceived size of a subsequent test stimulus. Large adapting stimuli cause the test to appear smaller than its veridical size and vice versa. Here, we investigated whether size adaptation is susceptible to attentional modulation. First, we measured the magnitude of adaptation aftereffects for a size discrimination task. Then, we compared these aftereffects (on average 15–20%) with those measured while participants were engaged, during the adaptation phase, in one of the two highly demanding central visual tasks: Multiple Object Tracking (MOT) or Rapid Serial Visual Presentation (RSVP). Our results indicate that deploying visual attention away from the adapters did not significantly affect the distortions of perceived size induced by adaptation, with accuracy and precision in the discrimination task being almost identical in all experimental conditions. Taken together, these results suggest that visual attention does not play a key role in size adaptation, in line with the idea that this phenomenon can be accounted for by local gain control mechanisms within area V1.

## Introduction

Achieving a reliable representation of the surrounding space is one of the most critical tasks that the animals’ brain (including humans) has to accomplish. For example, accurate judgment of the size or distance of the objects in the environment is critical for survival as it allows to successfully interact with them. Accordingly, much research has been dedicated to unveil the brain mechanisms underpinning objects’ size perception. Nevertheless, the exact mechanisms that underlie size perception are yet poorly understood (Schwarzkopf et al., 2010).

One important characteristic of size perception demonstrated by many studies is its susceptibility to contextual effects. For example, in the Ebbinghaus illusion (Massaro and Anderson, 1971), two identical circles surrounded by large and small stimuli are perceived as having different sizes. The stimulus surrounded by large flankers appears smaller compared to the other. Mario Ponzo reported another well-known illusion (i.e., Ponzo Illusion), where two identical horizontal lines drawn across a pair of converging lines, one on top and one below, appear to have different lengths; with the one above looking longer than the one below (Leibowitz et al., 1969).

In light of this evidence showing robust contextual modulations of the perceived objects’ size, it has been proposed that stimulus size is represented in high-level, associative visual areas such as occipitotemporal cortex where multiple cues related to the objects’ identity are combined together (Eger et al., 2008; Konkle and Oliva, 2012). Another related line of evidence supporting this idea is that objects’ size is coded in terms of their abstract, real-world as opposed to retinal size, i.e., an elephant is judged to be bigger than a table even when both are presented as images with identical sizes (Konkle and Oliva, 2012; Henik et al., 2017). However, a series of recent studies (Murray et al., 2006; Sperandio et al., 2012; Pooresmaeili et al., 2013) suggested instead that size perception may occur at even earlier stages of visual processing, for instance at the level of the primary visual cortex (area V1). In a fMRI study, Murray et al. (2006) leveraged pictorial cues to manipulate the perceived position of two identical objects in depth (i.e., Ponzo illusion) while recording participants brain activity in area V1. The results show that V1 activation scaled with the perceived size of the objects despite their physical size remaining constant. This suggests that V1 activation combines the incoming retinal signals representing the physical size of the objects with feedbacks from higher-level areas processing objects’ position in depth (see also Fang et al., 2008). Similarly, Sperandio et al. (2012) measured V1 activity when participants perceived afterimages of different sizes caused by the projection of the same object on surfaces that were at different viewing distances. The results clearly indicated that V1 activity changed in accordance with the perceived and not the physical size of the afterimage.

Despite these studies suggesting a key role of V1 in constructing an internal representation of objects size, the use of perspective pictorial cues to manipulate perceived size may lead to the involvement of extra-striate cortex where such cues are most reliably coded (Trotter and Celebrini, 1999; Andersson et al., 2007). To overcome such possible effects, size perception has been recently investigated by employing a contextual manipulation that does not use any perspective cue, i.e., perceptual adaptation (Pooresmaeili et al., 2013; Tonelli et al., 2017). After a prolonged exposure to a stimulus of a given size (adapter), the perceived size of a subsequent stimulus presented in the same position of the visual field (test) was found to be robustly distorted according to the classical rebound adaptation effects: larger adapting stimuli caused the test to appear smaller and vice versa. The computational model proposed to account for such adaptation aftereffects was based on a gain control mechanism in which perceived size is influenced by a mechanistic combination of inhibitory and excitatory cortical signals induced by the adapter and test stimuli. In detail, after a sustained presentation of the adapting stimulus, the activity of the V1 regions representing the adapter edges is reduced, changing the gain of responses of the nearby regions of the striate cortex. When the target stimulus is subsequently presented, the typical cortical activation to this stimulus is distorted by the gain modulation produced by the adapter. In conclusion, the distortion of objects sizes was proposed to arise from gain control mechanisms occurring locally at the level of area V1, whereby the area V1 neural activity matched the perceived, and not the physical size of the object. Although local gain modulation mechanisms at the level of area V1 could sufficiently explain size adaptation effects observed in these previous studies (Sperandio et al., 2012; and in particularly Pooresmaeili et al., 2013), it is still possible that top-down, feedback mechanisms arising from extra-striate cortex (area V2, V3, or V4), higher-level visual areas (such as lateral occipital cortex, area LO) or the fronto-parietal attentional network also influence the strength of the putative local interactions (Schwabe et al., 2006; Gilbert and Li, 2013). To investigate this possibility, Sperandio et al. (2012) tested BOLD responses in retinotopic visual areas beyond area V1. BOLD responses in the areas V2 and V3 showed no modulation in relation to the size of afterimages, which casts doubt on the involvement of these extra-striate areas in the representation of objects’ perceived size. Pooresmaeili et al. (2013), however, found that the strongest size adaptation effect across all tested conditions (i.e., with the adapter being larger, identical, or smaller in size than the test stimulus) occurred in areas V1–V3, whereas in area V4 only a trend was found that did not reach statistical significance. In area LO (Lateral Occipital Cortex) the adaptation effect was only observed when the adapter and the test had an identical size, which is in line with the role of this area in object categorization (Grill-Spector et al., 1999). Therefore, in both studies, the most robust correlates of perceived size were found in area V1. Is it possible that these correlates of size perception at the level of area V1 reflect mechanisms that are stirred by the allocation of visual attention? This is a plausible question given that attention can influence almost all aspects of visual processing. As demonstrated by a host of previous research, the deployment of spatial attention not only increases sensitivity, shortens reaction times and induces a more accurate performance (Posner et al., 1980; Dosher et al., 1986; Yeshurun and Carrasco, 1998), but also alters stimulus appearance (for a review see Gobell and Carrasco, 2005). Indeed, attention has been reported to increase apparent contrast (Carrasco et al., 2004), spatial frequency (Lamb and Yund, 1996), motion coherence (Liu et al., 2006), and perceived speed (Anton-Erxleben et al., 2013). Moreover, the relationship between visual attention and the perception of object size has been also directly investigated. On one hand, it has been shown that attention alters the perceived objects size (Anton-Erxleben et al., 2007), while on the other hand, objects’ size has been demonstrated to interact with the way that visual attention is allocated (Collegio et al., 2019).

Many influential models of attention maintain that modulation of visual processing by attention relies on top-down feedback signals that originate from a fronto-parietal network (Corbetta and Shulman, 2002) and influence upstream visual areas such as area V1, possibly through a gain-control mechanism (Reynolds and Chelazzi, 2004). Inspired by this theoretical framework, some studies have employed manipulations of visual attention as a means to investigate whether the perceived objects’ size is genuinely coded at the level of the primary visual areas or it originates from feedback pathways conveying contextual information from higher visual areas. For instance, exploiting a paradigm similar to Murray et al. (2006) and Fang et al. (2008) reported that the focus of visual attention alters the representation of objects size in V1. Specifically, while spatial distribution of V1 activity represented the perceived rather than physical size of the stimuli – activities induced by a stimulus perceived to be located further away yielded a more eccentric representation than those evoked by a stimulus perceived as being closer – directing attention elsewhere significantly reduced this effect. This result is consistent with the idea that diverting the focus of visual attention attenuates the top-down influence of higher visual areas on V1, thereby dampening V1’s capacity to integrate 3D depth cues. In a complementary study, Kreutzer et al. (2015) reported a significant role of selective attention in contextual modulation of object size perception even when they did not entail high-level perspective cues. Taken together, the picture emerging from these latter studies suggests that the local gain control mechanisms proposed by Pooresmaeili et al. (2013) might in fact result from long-distance feedback signals originiating from elsewhere in the visual hierarchy.

In the current study, we try to resolve this controversy by directly testing whether visual attention mediates the visual size adaptation effects observed in our previous work (Pooresmaeili et al., 2013). We asked participants to perform a size discrimination task in the periphery of the visual field while, during visual adaptation, they performed one of the two sustained attentional tasks (tested in separated sessions) at fovea. These tasks comprised either a rapid serial visual presentation (RSVP – Broadbent and Broadbent, 1987), or a multiple object-tracking task (MOT – Pylyshyn and Storm, 1988) engaging temporal or spatio-temporal attention, respectively. Participants’ performance was assessed in terms of accuracy (Point of Subjective Equality: PSE) as well as precision (Weber Fractions: WFs), that is the discrimination threshold normalized by the PSE. If the effects of adaptation on the processing of objects’ size tap on automatic, pre-attentive processes, withdrawing attention from the adapter location and allocating it elsewhere should not affect subjects’ size estimations. Conversely, the involvement of attention-dependent mechanisms will predict a reduction or disappearance of size adaptation effects when attention is diverted away from the adapter.

## Materials and Methods

### Participants

Nine healthy adults (three males, six females, average age: 27.7 years of age – *SD* = 0.97) with normal or corrected to normal vision participated in the experiments. All participant have been recruited from the faculty of psychology of the local university and have not been compensated to participate in the study. The local ethics committee (Comitato Etico Pediatrico Regionale, Azienda Ospedaliera-universitaria Meyer, Florence) approved all experimental procedures. The study was carried out in accordance with the Declaration of Helsinki and each participant gave informed consent before participation.

### Stimuli

**FIGURE 1:**
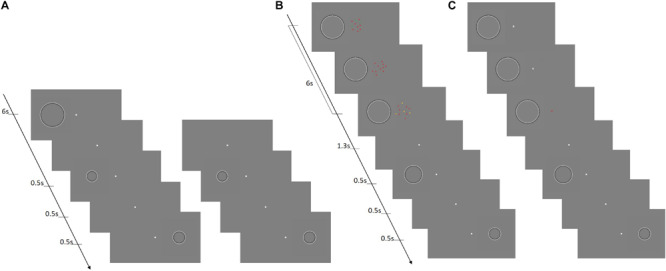
The size discrimination task with and without attentional manipulation. **(A)** The basic size discrimination task without attentional manipulation. The left and right panels illustrate the adaptation and baseline conditions, respectively. In adaptation condition, first an adapter stimulus was presented on the left of the screen for a duration of 6 s. Subsequently, a test stimulus appeared in the same location followed by a reference stimulus, presented symmetrically on the right side of the screen. The right panel illustrates the sequence of stimuli for the baseline condition where no adapter was presented. **(B)** The first attentional manipulation comprised a MOT condition, in which the sequence of presentation of the stimuli for the size discrimination was the same as the adaptation condition depicted in **(A)** (on the left), with the only exception that during the adaptation phase the stimuli used for the MOT task were presented at the fovea. **(C)** The second type of attentional manipulation consisted of a RSVP condition (presentation sequence identical to left in **A**). In this condition, during the adaptation phase the stimuli used for the RSVP task were presented at the fovea.

All visual stimuli were generated in MATLAB, using the Psychophysics Toolbox version extensions and displayed on a Mitsubishi Diamond Plus 220 monitor (resolution of 1,280 × 1,024 corresponding to 41°× 31.2° from participants’ viewing distance of 57 cm) with a refresh rate of 85 Hz. In the size discrimination task, stimuli consisted of Craik–O’Brien–Cornsweet circles defined by high-pass Gaussian filters (with a 50% cutoff at spatial frequency of 0.5 cyc/deg). The polarity of the stimuli was reversed at a rate of 10 Hz to avoid afterimages and the presentation time was always set to 500 ms (see Figure 1A). Stimuli for the MOT task consisted of 12 dots with a diameter of 0.4°. The dots moved at a speed of about 2 deg/sec in straight lines, and when colliding with other dots bounced appropriately with their motion constrained within a central invisible circular area of 5° of radius (Figure 1B). In the rapid serial visual presentation task (RSVP), participants were presented with small red or white alphabetic letters subtending 2°× 2° displayed at the center of the screen at a rate of about 10 Hz within the 6 s of the adaptation phase (Figure 1C).

### Procedures

#### Size Discrimination

A sequences of two Craik–O’Brien–Cornsweet circles were displayed at two diametrically opposite positions along the monitor horizontal midline at an eccentricity of 10° from the monitor center. In each trial, the size (diameter) of the reference stimulus (displayed on the right) was kept constant at 5° whilst the size of the test stimulus (displayed on the left) varied according to an adaptive staircase QUEST (Watson and Pelli, 1983) with the test size constrained to appear within + –50% of the size of the reference (Figure 1). The participants’ task consisted of indicating which stimulus was larger by pressing a key on a PC keyboard. In the adaptation trials, the presentation of the test and reference stimuli was preceded by a large Craik–O’Brien–Cornsweet circle (10° diameter so that the physical size of the adapter was always larger than the test) displayed on the left-hand side. At the end of the adaptation phase (6 s) the adapter disappeared and the sequence of test and reference stimulus started after 1,300 ms (i.e., inter-trial interval, ITI = 1,500 ms). Each time the discrimination task was carried out, each participant completed 100 trials.

#### Rapid Serial Visual Presentation (RSVP)

In this condition, during adaptation, a sequence of red and white letters was presented in the center of the screen. Participant had to indicate whether the number of red letters was more or less than 10 by pressing a key within a 1.3 s interval from the sequence offset (all slower responses were excluded from the analyses). Each participant performed 100 trials each time the task was performed.

#### Multiple Object Tracking (MOT)

In this condition, during adaptation, participants were presented with 12 randomly moving dots: nine red (distractors) and three green (targets). Participants had 2 s to lock their attention on the target dots, and then these turned to red and became undistinguishable from the distractors. At the end of this tracking phase lasting 4 s, four out of the twelve dots became orange and the task was to indicate whether one of the orange dots was green at the beginning by pressing a key within 1.3 s. Again, all slower responses were excluded from the following analyses. Each participant performed 100 trials each time the task was performed.

At the beginning of the experiment, participants’ performance for RSVP and MOT task was measured for several rates of letter presentation (RSVP) and dots speed (MOT). The aim of this procedure was to adjust the difficulty of the two tasks for each participant so that a correct rate of around 70–75% could be achieved for each individual. This procedure allows assessing an increase or a decrease in performance when these tasks were performed during the presentation of the adapting stimuli and concurrent with the size discrimination task.

Subsequently, each participant performed the task of discrimination in baseline and after that where performed three conditions of adaptation in random order between the adaptations with or without attending the MOT or RSVP stimuli and whether or not had to paid attention to the attentional stimuli.

## Results

**FIGURE 2:**
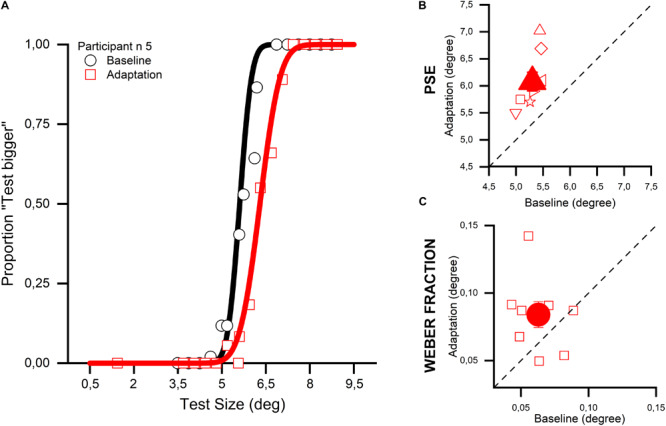
Size adaptation effect in the absence of attentional manipulation. **(A)** The psychometric function of one participant (number 5) is shown for the baseline (in black) and the adaptation condition (in red). Adaptation to a 10° stimulus with a reference of 5° produced a rightward shift of the curve. This shift indicates that the adaptation produced a reduction of the perceived size of the test stimulus, because the adaptive algorithm used during the discrimination task needed to increase the size of the test stimulus so that it was perceived identical to the reference stimulus. **(B)** The big filled red triangle depicts the average PSE for all the participants, while the small empty symbols show the data for the single participant. On the x-axis the PSEs for the baseline condition are plotted, while on the y-axis the PSEs for the adaptation condition are shown. **(C)** The big filled red circle shows the average WF for all the participants, while the small empty symbols correspond to the data for the individual participants. On the x-axis the WFs for the baseline condition are plotted, while on the y-axis the WFs for the adaptation condition are shown.

In the first experiment (Exp. 1), we measured the effect of a sustained exposure to a large stimulus (adapter) on the perception of the size of a smaller stimulus subsequently displayed at the adapted location. We measured the physical size of the adapted stimulus (test) needed to make it appear as large as the reference stimulus displayed in a neutral location (PSEs) and compared them with those obtained when size discrimination was performed without adaptation. Figure 2A shows how the percentage of “test stimulus larger” responses varied relative to the physical size of the test stimulus for a representative subject. In the baseline condition (no-adaptation), estimates for the test stimulus were rather veridical with the PSEs close to 5° (i.e., physical size of the reference). However, when the presentation of the test stimulus was preceded by a large adapting stimulus, its perceived size was robustly compressed as shown by the rightward shift of the red psychometric function. All participants showed robust adaptation aftereffects (see Figure 2B) making the difference between the baseline and adaptation condition highly statistically significant (two-tailed paired-sample *t*-test, *t*_8_ = 6.204, p < 0.001, 95% CI [0.49, 1.07]), a result that replicates previous studies exploiting a similar paradigm (Pooresmaeili et al., 2013; Tonelli et al., 2017). Moreover, a close inspection of the psychometric functions shown in Figure 2A suggests that the slope of the curve of the adaptation condition (red) was shallower than that for the no-adaptation (black) condition. Such difference opens up the possibility that adaptation not only affects the *accuracy* of size estimates (as shown by shifts of the PSEs) but also their *precision* to yield lower thresholds to discriminate between the test and reference. To test this hypothesis, we performed an analysis where for each participant the precision of stimulus size discrimination was measured in terms of Weber Fractions (WFs – discrimination thresholds normalized by PSEs), in both the baseline and adaptation conditions (Figure 2C). WFs for the adaptation condition were found to be, on average, slightly higher than in the baseline condition indicating that the exposure to the adapting stimuli lowered the size discrimination sensitivity; a result at odds with the hypothesis that sensory adaptation is aimed to increase the discriminability of similar stimuli (Barlow’s efficient coding hypothesis, see Barlow, 1961; Simoncelli, 2003). However, due to the high variability amongst participants, this difference turned out to be just marginally significant (two-tailed paired-sample *t*-test, *t*_8_ = 1.843, *p* = 0.1, 95% CI [−0.005, 0.047]), thus a definitive statement cannot be made about this result at the present stage.

**FIGURE 3:**
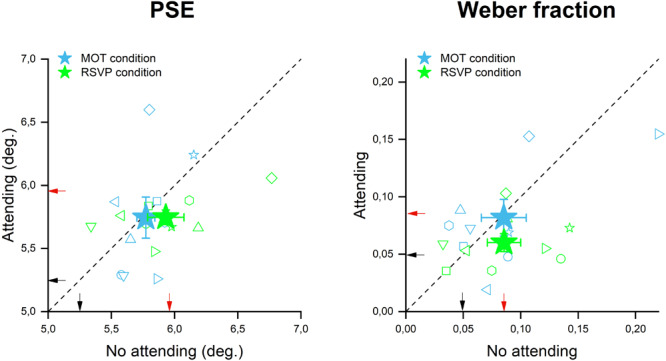
Size adaptation effect in the presence of attentional manipulation. The scatter plot on the left represents the PSEs during the no attending (x-axis) and attending (y-axis) conditions for both attentional tasks: MOT in cyan and RSVP in green. The filled stars are the average PSE across all participants, while the small empty symbols are the performance of each participant. The scatter plot on the right represent the WFs during the no attending (x-axis) and attending (y-axis) conditions for both the attentional tasks: MOT in cyan and RSVP in green. Also in this plot, the filled stars are the average across all participants (with error bar representing ±1 s.e.m), while the small empty symbols are the performance of each participant. The small black and red arrows indicate the PSEs and the WFs achieved in Exp 1 (in which no central stimuli were presented) for the baseline and adaptation condition respectively.

To assess the role of the focus of attention in the size adaptation phenomenon, we devised two new versions of Exp. 1 in which during the adaptation phase, participants were presented with a central stimulus corresponding to either a temporal (RSVP) or a spatio-temporal (MOT) attentional task. In one case (i.e., central attention condition), participants were required to perform a task on this central display while in the other case (no central attention condition) they passively viewed this display but performed no task on it. The rationale was to compare participants’ accuracy and precision in the size discrimination task between these two conditions, which were identical in terms of sensory loads but differed in terms of the allocation of attention. Figure 3 shows the participants’ accuracy and precision of size discrimination when a RSVP or MOT task was performed during adaptation. The perceived size of the test stimulus was quite similar when RSVP stimuli were displayed but subjects were instructed to ignore them (no attending- mean 5.9, *SD* = 0.4) relative to the condition in which RVSP had to be accomplished and thus attention was withdrawn from the adapting stimulus location (mean 5.74, *SD* = 0.17, see green stars in Figure 3A). This result suggests that shifting the focus of attention away from the adapting stimuli – via a temporal attentional task – did not significantly affect the amount of size adaptation induced by them. Indeed, both conditions (attending and not attending to the central RSVP task) turned out to be not significantly different from the adaptation condition of Exp. 1 where no central stimulus was displayed at all [a one-way ANOVA: *F*_(__2_,_16__)_ = 3.35, *p* = 0.07, ges = 0.127]. One possibility for this lack of interference between size adaptation and the deployment of attentional resources away from the adapters might be that the RSVP task did not involve any spatial processing, which is at the core of objects’ size perception. To test for this hypothesis, we replicated the previous experiment by using MOT as the central task as it implies a dynamic allocation of attention to a different spatial location, over time, to track multiple moving targets. However, even in this case, the magnitude of size adaptation indicated by the averaged PSEs achieved when participants were engaged with the central task (y axis position of cyan star in Figure 3A, mean 5.74, *SD* = 0.46) was found to be similar to the condition in which no central task was performed (x axis of the cyan star in Figure 3A, mean 5.77, *SD* = 0.2). This adaptation magnitude also turned out to be similar to those achieved in Exp. 1 when no central stimuli were displayed at all [a one-way ANOVA: *F*_(__2_,_16__)_ = 2.3, *p* = 0.31, ges = 0.137]. Taken together these results suggest that neither the increase of sensory load induced by the mere presentation of the central stimuli (the no attending to central task condition), nor the shift of attentional resources away from the adapters (attending to the central task condition) significantly affect the magnitude of size adaptation aftereffects.

However, even if sensory or attentional load did not affect the accuracy of stimulus size estimates (PSEs); they might still have significantly affected discrimination precision. To test this hypothesis, we measured WFs for the “not attending” and “attending” to the central task conditions for both RSVP and MOT task. WFs measured with the MOT as a central task (Figure 3B, in cyan) were found to be almost identical between the “attending” (mean 0.08, *SD* = 0.04) and “not attending” (mean 0.08, *SD* = 0.05) to the central task conditions with these values also being very similar to those achieved in Exp. 1 [a one-way ANOVA: *F*_(__2_,_16__)_ = 0.02, *p* = 0.97, ges = 0.001]. On the other hand, WFs measured when participants performed RSVP as a central task turned out to be slightly smaller in the “attending” condition (see the green star Figure 3B) than in the “not attending” and the no-central stimuli Exp. 1 condition. However, due to the rather substantial variability amongst participants, this difference was not statistically significant [a one-way ANOVA: *F*_(__2_,_16__)_ = 2.9, *p* = 0.09, ges = 0.135; no attending mean 0.08, *SD* = 0.02; attending mean 0.06, *SD* = 0.04].

**FIGURE 4:**
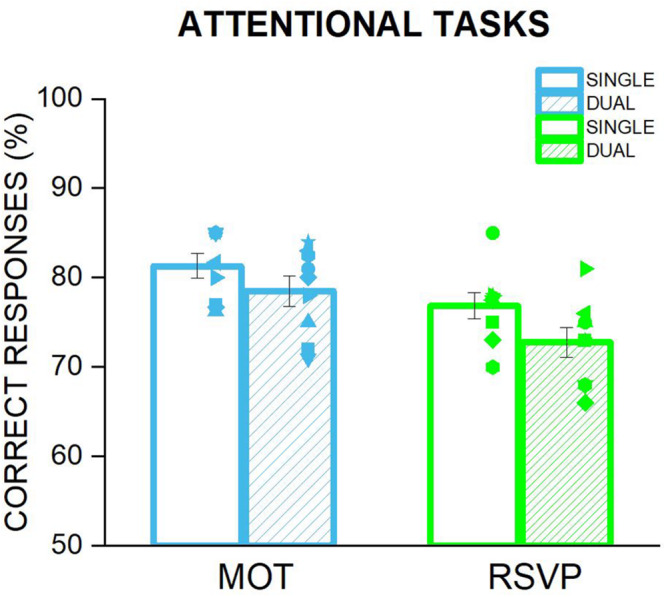
Comparison of the performance in MOT and RSVP tasks when performed alone and when undertaken during the adaptation phase of the size discrimination task. Each bar represents the average of correct responses for the two attentional tasks: MOT in cyan and RSVP in green. The empty bars are the percentage of correct responses when the task was performed alone, i.e., not during the adaptation of the discrimination task. This condition has been used to adjust the difficulty of the two tasks for each participant, so that it would be possible to achieve a correct rate of around 70–75%. The striped bars depict the average performance in the attending condition during the size discrimination task, i.e., the participants performed the attentional task at the fovea, while the adapter stimulus was presented in the left. Symbols are the performance of each participant whilst error bar represent ±1 s.e.m.

One possibility to account for the lack of a significant change accuracy or precision when the focus of attention is manipulated might be that participants preserved adaptation aftereffects by deploying a negligible amount of their attentional resources to the central task either voluntarily or due to the peripheral flickering of the adapters automatically capturing their attention. In other words, it might be that performance in the size discrimination task was preserved at the cost of the central task. If so, participants’ performance in the RSVP or MOT task when they were performed on their own should be higher than when they were performed during the size discrimination task. However, as shown in Figure 4, the percentage of correct responses for the RSVP or the MOT task when the central stimuli were presented simultaneously with the peripheral adapters were almost identical to those measured when these tasks were performed alone (RSVP: *t* = 1.62, *p* = 0.12; MOT: *t*_8_ = 1.35, *p* = 0.2). These results clearly rule out the possibility that attention did not affect size adaptation because it was not sufficiently engaged by the central task. Therefore a an insufficient deployment of attentional resources on the central tasks cannot account for the similar magnitude of adaptation aftereffects found for the condition in which subjects were engaged in a central task and those where they were not.

## Discussion

In this study, we investigated the role of attention in mediating size adaptation aftereffects, i.e., distortions of perceived size of visual objects induced by the relative size of stimuli previously displayed in the same area of the visual field. Our results replicate previous findings showing that as a consequence of a prolonged exposure to a given visual stimulus, perceived size of the patterns subsequently presented in that area are distorted as follows: larger adapting stimuli cause the test to appear smaller than its veridical size and vice versa (Pooresmaeili et al., 2013; Tonelli et al., 2017). However, the main goal of the present study was to assess whether, and to what extent, attentional manipulations (such as deploying attention away from the adapters during the adaptation phase) affect size adaptation aftereffects. The results clearly demonstrated that attention *did not* affect either the accuracy or the precision of subject’s performance in the discrimination task, suggesting that size adaptation occurs independently from attentional mechanisms. In particular, neither the PSEs (physical size of the adapted stimulus perceived as large as the reference) nor the Weber Fractions (the just noticeable physical difference between the test and reference stimulus) of subjects was significantly changed by engaging in a central perceptual task during exposure to adapting stimuli.

We used two different central attentional tasks (tested in separate sessions), a RSVP mainly engaging temporal attention and a MOT task primarily requiring spatio-temporal attentional resources to track moving stimuli. The rationale was to test for a possible role of “similarity” between the type of attentional resources engaged in the central task and the perceptual processes engaged by size adaptation. The reasoning we followed here to use two types of attentional manipulation was inspired by previous cross-modal studies investigating whether attentional resources for different sensory modalities are shared or independent. Several studies support the latter hypothesis by showing that subjects’ performance in a given perceptual task (i.e., visual or auditory) remained unchanged when they concurrently performed a second task in a different sensory modality. However, as brilliantly demonstrated by Wahn and König (2015a, b), this independence only occurred when the primary and the secondary task engaged two different types of attention (i.e., object-based vs. spatial). On the other hand, if the primary and the secondary task both engaged the same type of attentional resources (i.e., spatial attention), subjects’ performance in one task impaired performance in the other task suggesting shared attentional resources across different sensory modalities.

Based on these previous observations, we employed two tasks, which involved different degrees of similarity with the type of putative attentional mechanisms that could underlie size perception. Neither the RSVP task that engaged attention across time, nor the MOT task which relied on allocation of attention across space and time yielded significant changes in size adaptation aftereffects (see Figure 3) pointing to an almost complete independence of size adaptation from both temporal and spatio-temporal attentional processes. Importantly, we also ruled out the possibility that size discrimination performance was preserved *at the cost* of the central task as subject’s performance in both RSVP and MOT tasks during adaptation phase of the size discrimination task did not significantly differ from when they were performed alone (see for details Figure 4). Moreover, we were able to disentangle the effects of attentional deployment from those induced by a change in the sensory load, due to the mere presence of the stimuli of the central task. To this end, we measured the accuracy and precision of the size discrimination task under two conditions. In one condition, subjects were required to attend to the central stimuli while in the other condition they were instructed not to do so (see Figure 3). However, keeping the amount of sensory information the same, we found that engaging subjects in a central attentional task during adaptation did not yield any significant effect pointing again to the independence of mechanisms underlying size adaptation from attention.

Interestingly, although attention has been reported to affect many aspects of visual perception, such as apparent contrast (Carrasco et al., 2004), spatial frequency (Lamb and Yund, 1996), motion coherence (Liu et al., 2006), or perceived speed (Anton-Erxleben et al., 2013) and to robustly affect population receptive fields (pRFs) in all areas along the visual hierarchy (Klein et al., 2014), conflicting results have been reported regarding its role in mediating adaptation aftereffects. On one side, attention has been reported to affect adaptation to high level stimuli such as faces (Rhodes et al., 2011) or body size (Stephen et al., 2018). On the other side, however, adaptation aftereffects for low-level visual features have been reported to be attention-independent. For example, Morgan (2012) reported a significant adaptation of stimulus perceived velocity that was completely independent of the amount of attention deployed to the test stimulus. The same author also demonstrated that motion aftereffects induced by adaptation occur independently from subjects’ attentional load during the task. In other words, Morgan‘s results support the idea that visual adaptation for features that are encoded at the early stages of visual processing hierarchy, might be primarily attention- independent. Size adaptation is likely to be one of these processes given that its aftereffects have been successfully modeled in terms of a simple gain control mechanism in which perceived size is retrieved through a combination of inhibitory and excitatory cortical signals induced by the adapter and test stimuli (Pooresmaeili et al., 2013).

However, not all the results in the literature on perception of objects’ size are in line with this interpretation. For example, it has been demonstrated that the representation of objects’ size in the primary visual area (V1) is attention-dependent (Fang et al., 2008). The authors showed that cortical activations to the same object depicted at two different “depth positions” of a rendered three-dimensional hallway (a version of the well-known Ponzo illusion; Leibowitz et al., 1969) differed according to the perceived object size (Murray et al., 2006). This perceptual illusion was, however, strongly attenuated when spatial attention was diverted away from the test stimuli. It is important to note that in this experiment perceived objects size was manipulated via complex 3D contextual information that are likely to tap on the feedback projections from extra-striate visual areas (involved in processing 3D pictorial cues) down to the V1. Given that these processes are, in turn, likely to be mediated by attentional mechanisms, the difference in the contextual information used in Fang et al. (2008) and Murray et al. (2006) studies (prospective 3D), and the ones employed in the present study (relative size of 2D objects) might explain the differences in obtained results. Lastly, we note another study, which reported a significant effect of spatial attention in mediating size adaptation without using prospective cues (Kreutzer et al., 2015). The role of attention was investigated by presenting a single adapting stimulus containing both a large and a small adapter, and requiring subjects to selectively direct their attention to one of the adapters before performing a size discrimination task. The results showed that the perceived size of a subsequently displayed test stimulus inversely covaried with the size of the attended adapter suggesting that attention mediates size adaptation aftereffects. However, the simultaneous presentation of flickering stimuli defining the large and the small adapters at a relatively close distance from each other might have made it difficult for the subjects to deploy selectively attention to one of the two adapters. In line with this, the reported size of adaptation aftereffects was quite small (changes of perceived size induced by the small adapter were about 3%) as well as asymmetrical (large adapter not affecting the perceived size of the test stimulus), contrary to other reports investigating size adaptation (Pooresmaeili et al., 2013; Tonelli et al., 2017). Future studies are needed to test these speculations and clarify in which conditions attention mediates size adaptation and in which conditions size adaptation aftereffects are attention-independent.

## Data Availability Statement

The datasets generated for this study are available at the following link http://doi.org/10.5281/zenodo.3831328.

## Ethics Statement

The studies involving human participants were reviewed and approved by Comitato Etico Pediatrico Regionale, Azienda Ospedaliero-universitaria Meyer, Florence (FI). Written informed consent to participate in this study was provided by the patient/participants.

## Author Contributions

AT and RA contributed conception, design of the study, and wrote the first draft of the manuscript. AT collected the data and performed the statistical analysis. All authors contributed to manuscript revision, read, and approved the submitted version.

## Conflict of Interest

The authors declare that the research was conducted in the absence of any commercial or financial relationships that could be construed as a potential conflict of interest.

## References

[B1] Andersson F. Joliot M. Perchey G. Petit L. (2007). Eye position-dependent activity in the primary visual area as revealed by fMRI. *Hum. Brain Mapp.* 28 673–680. 10.1002/hbm.20296 17089375 PMC6871435

[B2] Anton-Erxleben K. Henrich C. Treue S. (2007). Attention changes perceived size of moving visual patterns. *J. Vis.* 7 1–9. 10.1167/7.11.5 17997660

[B3] Anton-Erxleben K. Herrmann K. Carrasco M. (2013). Independent effects of adaptation and attention on perceived speed. *Psychol. Sci.* 24 150–159. 10.1177/0956797612449178 23241456 PMC3570681

[B4] Barlow H. B. (1961). “Possible principles underlying the transformations of sensory messages,” in *Sensory Communication*, (Cambridge, MA: The MIT Press), 216–234. 10.7551/mitpress/9780262518420.003.0013

[B5] Broadbent D. E. Broadbent M. H. P. (1987). From detection to identification: response to multiple targets in rapid serial visual presentation. *Percept. Psychophys.* 42 105–113. 10.3758/bf03210498 3627930

[B6] Carrasco M. Ling S. Gobel J. Fuller S. Read S. (2004). Attention alters appearance in early vision: contrast sensitivity, spatial resolution, and color saturation. *J. Vis.* 4:67.

[B7] Collegio A. J. Nah J. C. Scotti P. S. Shomstein S. (2019). Attention scales according to inferred real-world object size. *Nat. Hum. Behav.* 3:40. 10.1038/s41562-018-0485-2 30932061

[B8] Corbetta M. Shulman G. L. (2002). Control of goal-directed and stimulus-driven attention in the brain. *Nat. Rev. Neurosci.* 3:201. 10.1038/nrn755 11994752

[B9] Dosher B. A. Sperling G. Wurst S. A. (1986). Tradeoffs between stereopsis and proximity luminance covariance as determinants of perceived 3D structure. *Vis. Res.* 26 973–990. 3750879 10.1016/0042-6989(86)90154-9

[B10] Eger E. Ashburner J. Haynes J.-D. Dolan R. J. Rees G. (2008). fMRI activity patterns in human LOC carry information about object exemplars within category. *J. Cogn. Neurosci.* 20 356–370. 10.1162/jocn.2008.20019 18275340 PMC2832116

[B11] Fang F. Boyaci H. Kersten D. Murray S. O. (2008). Attention-dependent representation of a size illusion in human V1. *Curr. Biol.* 18 1707–1712. 10.1016/j.cub.2008.09.025 18993076 PMC2638992

[B12] Gilbert C. D. Li W. (2013). Top-down influences on visual processing. *Nat. Rev. Neurosci.* 14:350. 10.1038/nrn3476 PMC3864796 23595013

[B13] Gobell J. Carrasco M. (2005). Attention alters the appearance of spatial frequency and gap size. *Psychol. Sci.* 16 644–651. 10.1111/j.1467-9280.2005.01588.x 16102068

[B14] Grill-Spector K. Kushnir T. Edelman S. Avidan G. Itzchak Y. Malach R. (1999). Differential processing of objects under various viewing conditions in the human lateral occipital complex. *Neuron* 24 187–203. 10.1016/S0896-6273(00)80832-80836 10677037

[B15] Henik A. Gliksman Y. Kallai A. Leibovich T. (2017). Size perception and the foundation of numerical processing. *Curr. Dir. Psychol. Sci.* 26 45–51.

[B16] Klein B. P. Harvey B. M. Dumoulin S. O. (2014). Attraction of position preference by spatial attention throughout human visual cortex. *Neuron* 84 227–237. 25242220 10.1016/j.neuron.2014.08.047

[B17] Konkle T. Oliva A. (2012). A real-world size organization of object responses in occipitotemporal cortex. *Neuron* 74 1114–1124. 10.1016/j.neuron.2012.04.036 22726840 PMC3391318

[B18] Kreutzer S. Fink G. R. Weidner R. (2015). Attention modulates visual size adaptation. *J. Vis.* 15:10. 10.1167/15.15.10 26575196

[B19] Lamb M. R. Yund E. W. (1996). Spatial frequency and attention: effects of level-, target-, and location-repetition on the processing of global and local forms. *Percept. Psychophys.* 58 363–373. 8935897 10.3758/bf03206812

[B20] Leibowitz H. Brislin R. Perlmutrer L. Hennessy R. (1969). Ponzo perspective illusion as a manifestation of space perception. *Science* 166 1174–1176. 10.1126/science.166.3909.1174 17775578

[B21] Liu T. Fuller S. Carrasco M. (2006). Attention alters the appearance of motion coherence. *Psychon. Bull. Rev.* 13 1091–1096. 17484441 10.3758/bf03213931

[B22] Massaro D. W. Anderson N. H. (1971). Judgmental model of the ebbinghaus illusion. *J. Exp. Psychol.* 89 147–151. 10.1037/h0031158 5569620

[B23] Morgan M. J. (2012). Motion adaptation does not depend on attention to the adaptor. *Vis. Res.* 55 47–51. 10.1016/j.visres.2011.12.009 22245710 PMC4135072

[B24] Murray S. O. Boyaci H. Kersten D. (2006). The representation of perceived angular size in human primary visual cortex. *Nat. Neurosci.* 9 429–434. 16462737 10.1038/nn1641

[B25] Pooresmaeili A. Arrighi R. Biagi L. Morrone M. C. (2013). Blood oxygen level-dependent activation of the primary visual cortex predicts size adaptation illusion. *J. Neurosci.* 33 15999–16008. 10.1523/JNEUROSCI.1770-13.2013 24089504 PMC4888977

[B26] Posner M. I. Snyder C. R. Davidson B. J. (1980). Attention and the detection of signals. *J. Exp. Psychol. Gen.* 109:160. 7381367

[B27] Pylyshyn Z. W. Storm R. W. (1988). Tracking multiple independent targets: evidence for a parallel tracking mechanism. *Spat. Vis.* 3 179–197. 3153671 10.1163/156856888x00122

[B28] Reynolds J. H. Chelazzi L. (2004). Attentional modulation of visual processing. *Annu. Rev. Neurosci.* 27 611–647. 15217345 10.1146/annurev.neuro.26.041002.131039

[B29] Rhodes G. Jeffery L. Evangelista E. Ewing L. Peters M. Taylor L. (2011). Enhanced attention amplifies face adaptation. *Vis. Res.* 51 1811–1819. 21704059 10.1016/j.visres.2011.06.008

[B30] Schwabe L. Obermayer K. Angelucci A. Bressloff P. C. (2006). The role of feedback in shaping the extra-classical receptive field of cortical neurons: a recurrent network model. *J. Neurosci.* 26 9117–9129. 16957068 10.1523/JNEUROSCI.1253-06.2006 PMC6674516

[B31] Schwarzkopf D. Song C. Rees G. (2010). The surface area of human V1 predicts the subjective experience of object size. *Nat. Neurosci.* 14 28–30. 10.1038/nn.2706 21131954 PMC3012031

[B32] Simoncelli E. P. (2003). Vision and the statistics of the visual environment. *Curr. Opin. Neurobiol.* 13 144–149. 10.1016/S0959-4388(03)00047-43 12744966

[B33] Sperandio I. Chouinard P. A. Goodale M. A. (2012). Retinotopic activity in V1 reflects the perceived and not the retinal size of an afterimage. *Nat. Neurosci.* 15 540–542. 10.1038/nn.3069 22406550

[B34] Stephen I. D. Sturman D. Stevenson R. J. Mond J. Brooks K. R. (2018). Visual attention mediates the relationship between body satisfaction and susceptibility to the body size adaptation effect. *PLoS One* 13:e0189855. 10.1371/journal.pone.0189855 29385137 PMC5791942

[B35] Tonelli A. Cuturi L. F. Gori M. (2017). The influence of auditory information on visual size adaptation. *Front. Neurosci.* 11:594. 10.3389/fnins.2017.00594 29114201 PMC5660698

[B36] Trotter Y. Celebrini S. (1999). Gaze direction controls response gain in primary visual-cortex neurons. *Nature* 398 239–242. 10.1038/18444 10094046

[B37] Wahn B. König P. (2015a). Audition and vision share spatial attentional resources, yet attentional load does not disrupt audiovisual integration. *Front. Psychol.* 6:1084. 10.3389/fpsyg.2015.01084 26284008 PMC4518141

[B38] Wahn B. König P. (2015b). Vision and haptics share spatial attentional resources and visuotactile integration is not affected by high attentional load. *Multisens. Res.* 28 371–392. 10.1163/22134808-22132482 26288905

[B39] Watson A. B. Pelli D. G. (1983). QUEST: a bayesian adaptive psychometric method. *Percept. Psychophys.* 33 113–120. 6844102 10.3758/bf03202828

[B40] Yeshurun Y. Carrasco M. (1998). Attention improves or impairs visual performance by enhancing spatial resolution. *Nature* 396:72. 10.1038/23936 PMC3825508 9817201

